# Intra-Abdominal Pressure Measurements in Term Pregnancy and Postpartum: An Observational Study

**DOI:** 10.1371/journal.pone.0104782

**Published:** 2014-08-12

**Authors:** Anneleen S. E. Staelens, Stefan Van Cauwelaert, Kathleen Tomsin, Tinne Mesens, Manu L. N. Malbrain, Wilfried Gyselaers

**Affiliations:** 1 Dept. Medicine and Life Sciences, Hasselt University, Hasselt, Belgium; 2 Dept. Obstetrics & Gynaecology, Ziekenhuis Oost Limburg, Genk, Belgium; 3 Dept. of Intensive Care, Ziekenhuis Netwerk Antwerpen, ZNA Stuivenberg, Antwerp, Belgium; University of Tennessee Health Science Center, United States of America

## Abstract

**Objective:**

To determine intra-abdominal pressure (IAP) and to evaluate the reproducibility of IAP-measurements using the Foley Manometer Low Volume (FMLV) in term uncomplicated pregnancies before and after caesarean section (CS), relative to two different reference points and to non-pregnant values.

**Design:**

Observational cohort study.

**Setting:**

Secondary level referral center for feto-maternal medicine.

**Population:**

Term uncomplicated pregnant women as the case-group and non-pregnant patients undergoing a laparoscopic assisted vaginal hysterectomy (LAVH) as control group.

**Methods:**

IAP was measured in 23 term pregnant patients, before and after CS and in 27 women immediately after and 1 day after LAVH. The midaxillary line was used as zero-reference (IAP_MAL_) in all patients and in 13 CS and 13 LAVH patients, the symphysis pubis (IAP_SP_) was evaluated as additional zero-reference. Intraobserver correlation (ICC) was calculated for each zero-reference. Paired student's *t*-tests were performed to compare IAP values and Pearson's correlation was used to assess correlations between IAP and gestational variables.

**Main outcome measures:**

ICC before and after surgery, IAP before and after CS, IAP after CS and LAVH.

**Results:**

The ICC for IAP_MAL_ before CS was lower than after (0.71 versus 0.87). Both mean IAP_MAL_ and IAP_SP_ were significantly higher before CS than after: 14.0±2.6 mmHg versus 9.8±3.0 mmHg (p<0.0001) and 8.2±2.5 mmHg versus 3.5±1.9 mmHg (p = 0.010), respectively. After CS, IAP was not different from values measured in the LAVH-group.

**Conclusion:**

IAP-measurements using FMLV is reproducible in pregnant women. Before CS, IAP is increased in the range of intra-abdominal hypertension for non-pregnant individuals. IAP significantly decreases to normal values after delivery.

## Introduction

Intra-abdominal pressure (IAP) was defined in 2006 by the World Society of Abdominal Compartment Syndrome (WSACS, www.wsacs.org) consensus definition as the steady state pressure concealed within the abdominal cavity [1], [2]. In general, a normal IAP varies from sub-atmospheric values to 7 mmHg in normal-weight individuals, with higher baseline levels in morbidly obese patients of about 9 to 14 mmHg [1], [3]. Intra-abdominal hypertension (IAH) is defined as a sustained increase in IAP≥12 mmHg and abdominal compartment syndrome (ACS) is defined as IAP>20 mmHg with new onset end-organ failure. Both IAH and ACS are associated with organ dysfunction, multisystem organ failure, high morbidity and mortality [1], [4]–[6].

To date, little is known about normal values of IAP during pregnancy, either in healthy or complicated pregnancies. In 1913, Paramore was the first to investigate IAP during pregnancy [7]. Transrectal measurement of IAP was higher in pregnant women compared to non-pregnant individuals, and values increased throughout the course of pregnancy. In a few case reports, it has been recently suggested that elevated IAP might play a role in some gestational complications, such as (pre)eclampsia [8]–[10] of which the hypothesis has been documented extensively [10]–[12].

IAP can be measured using a wide range of techniques [13]. Assessment of the IAP by intra-bladder pressure (IBP) measurement was first described by Kron et al. in 1984 [14] and is currently considered as the gold standard method because of its safety, simplicity and reliability. However, this technique has never been validated in pregnant subjects.

The aim of this study was to evaluate the reproducibility of IAP measurements in pregnant women before and after caesarean section (CS), using the Foley Manometer Low Volume technique (FMLV, Holtech Medical, Charlottenlund, Denmark) according to two different zero-reference points. Next, IAP values in term parturients were compared with values in non-pregnant women after gynecological surgery. Finally, the correlation between maternal-fetal parameters and IAP values was evaluated.

## Materials and Methods

### Ethics

This observational cohort study was approved by the ethical committees of Ziekenhuis Oost-Limburg (Genk, Belgium) and Hasselt University (Hasselt, Belgium) (U12/048). Oral and written informed consent was obtained from all patients.

### Patients

From March to April 2013, women with uncomplicated pregnancies admitted for elective primary or repeat caesarean section (CS) in Ziekenhuis Oost-Limburg Genk (Belgium) were invited to participate in the study. Exclusion criteria were age under 18 years, patients with nephritic syndrome, neurogenic or radiation bladder or abdominal masses. Next, twin pregnancies, patients in labor and patients with gestational complications such as preeclampsia, growth restriction and severe prematurity (<34 weeks) were also excluded. Women without comorbidity admitted for a laparoscopic assisted vaginal hysterectomy (LAVH) at Ziekenhuis Oost-Limburg Genk (Belgium) were included also as a control group.

### Data collection

Parameters registered for each patient were patient's height, current weight and age. Weight before pregnancy, gravidity, parity, estimated date of delivery according to crown rump length measurements during first-trimester ultrasound, fetal presentation and fetal birth weight were registered for every pregnant woman.

### Measurements

In the CS-group, IAP was measured by a single investigator at two time points and registered as the mean of three measurements each. Three consecutive measurements were performed in order to investigate the intra-observer variability. The first set of three measurements was performed one hour before CS; the second set of measurements was performed 24 hours after CS. The time interval between each intra-session measurement was approximately 10 minutes. Measurements were performed before any local or spinal anaesthesia.

**Figure 1:**
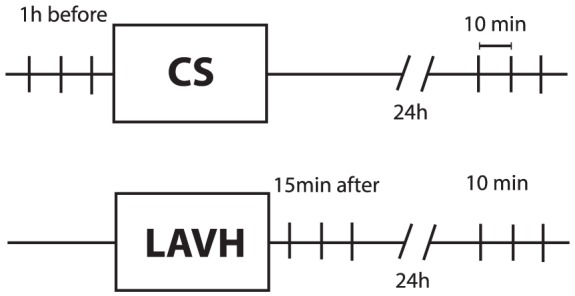
Study design for the CS group and the LAVH-group. Each vertical line stands for a single measurement. CS: caesarean section; LAVH: laparoscopic assisted vaginal hysterectomy.

In the control LAVH-group, IAP measurement was first performed after the surgery as the urinary bladder catheter is inserted during the operative procedure under general anaesthesia, because of departmental policy. Consequently in the LAVH-group, IAP was also measured in two sets of three measurements: one set 15 minutes after surgery and the other 24 hours after surgery. The study design is shown in figure 1. According to the recommendations by the World Society of the Abdominal Compartment Syndrome (WSACS, www.wsacs.org), IAP measurements were obtained with the patient in fully supine position, without head of bed (HOB) elevation and at the end of expiration [15]. Prior to each set of measurements, Richmond Agitations-Sedation Scale (RASS) score was assessed, which is a 10-point (−5 to +4) scale for levels of sedation (negative score) or agitation (positive score) in order to assess the level of consciousness and agitated behaviour [16].

**Figure 2:**
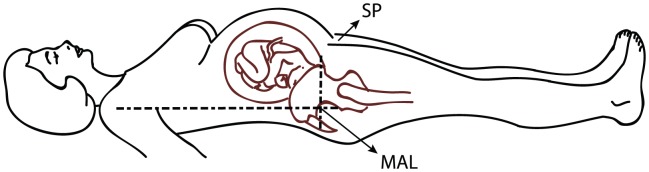
Anatomic orientations of the zero-reference points. SP: Symphysis pubis; MAL: Mid axillary line.

In both groups, the midaxillary line (IAP_MAL_) at the level of the iliac crest was used as zero-reference point for all measurements, as recommended by the WSACS [2], [15]. In a subgroup of 13 patients in the CS-group and 13 patients in the LAVH-group, a second zero-reference point using the symphysis pubis (IAP_SP_) was used. The anatomic orientation of both reference points is shown in figure 2. To assure each individual's zero-reference point at all time, a skin mark was applied in all subjects.

IBP was measured using a Foley Manometer Low Volume (FMLV) (Holtech Medical, Charlottenlund, Denmark) [17], which was placed one hour before CS in the CS-group and in the operating room just before the LAVH for the control group. In case of an empty urinary bladder or the presence of air-bubbles obstructing a continuous fluid column in the FMLV, 20 ml of 0.9% sterile sodium chloride solution was injected via the sample port. The urinary bladder catheter was clamped distal to the port to ensure an open pressure conductive fluid column. While measuring the IBP, the tone of the abdominal muscles was evaluated by palpation to ensure the absence of straining or spontaneous uterine contractions. When the meniscus of the fluid column in the FMLV had stabilized and oscillated according to the breathing pattern, the patient was asked to cease breathing at end-expiration. The corresponding value of IBP was registered in the database. Intra-abdominal hypertension is defined by an elevation in IAP≥12 mmHg. Grade I IAH is defined as IAP 12–15 mmHg, grade II as 16–20 mmHg, grade III as 21–25 mmHg and grade IV as IAP>25 mmHg [2]. Abdominal compartment syndrome is defined as IAP>20 mmHg that is associated with new organ dysfunction [2]. In case of the FMLV indicating a sub-atmospheric IBP, a value of −1 was registered in the database.

### Statistics

All data were tested for normality using the Shapiro-Wilk test. Values were expressed as mean with standard deviation (SD) when normally distributed and as median with interquartile range in case of non-normal distribution. Repeatability was evaluated by calculating the intra-class correlation coefficient (ICC) following a 2-way mixed model on absolute agreement for single measures. To compare the IAP values before and after the CS, a 2-sided, paired student's t-test was performed. Fisher's exact test was used to compare categorical variables. To evaluate correlation between IAP and gestational characteristics, the Pearson correlation coefficient (PCC) was calculated. When one of the parameters was dichotomous, the point-biserial correlation coefficient was used. Significant correlation was assessed at a nominal level of α = 0.05. For all calculations, the Statistical Package for the Social Sciences was used (SPSS Inc., Software version 20.0, IBM Corporations, New York, USA).

## Results

A total of 152 sets of 3 consecutive IAP measurements were conducted in 50 patients: 23 in the CS-group and 27 in the control LAVH-group. The demographics of each group are shown in table 1. Apart from a difference in age, there was no significant difference in length, (pre-gestational) weight and (pre-gestational) BMI between both groups. In the CS-group, the mean gestational age was 39w0d±0w4d. All patients had a RASS-score of 0 at each measurement.

**Table 1:** Demographics of the caesarean section (CS) group and the laparoscopic assisted vaginal hysterectomy group (LAVH).

	CS-group			LAVH-group			
Variable	N	mean	SD	N	mean	SD	*P*
*Age*	23	29.3	3.5	27	50.7	15.4	*<0.001*
*Length (cm)*	23	164.8	6.6	27	163.1	5.3	*0.33*
*Weight (kg)*	23	71.32	21.5	27	78.6	18.6	*0.21*
*BMI (kg/m^2^)*	23	26.1	7.3	27	29.6	7.1	*0.10*
*Weight before CS (kg)*	23	84.6	19.3				
*BMI before CS (kg/m^2^)*	23	31.0	6.4				
*Gestational age (weeks days)*	23	39.0	0.6				
*Fetal birth weight (g)*	23	3590.9	472.2				
*Fetal birth length (cm)*	23	49.4	2.8				
*Fetal birth weight percentile*	23	60.9	30.8				
*Smoking*	5			6			*0.79*
*Diabetes*	0			3			*0.09*
*Hypertension*	3			9			*0.08*
*Nulliparous*	4						
**Reason for CS**							
* Breech presentation*	7						
* Repeat CS*	16						
* Placenta praevia*	1						

Weight and BMI are pre-pregnancy for the CS-group. Numeric data are presented as mean with standard deviation (SD). P-values are calculated with an independent sample T-test for numeric data, and with a Fisher's exact test for dichotomous data.

The intra-class correlation (ICC) between the 3 consecutive measurements, before and after CS and after LAVH, is presented in table 2. The overall ICC is lower in the CS-group than in the LAVH-group (0.71–0.95 versus 0.96–0.98). Here, ICC before CS is lower than after CS (0.71 versus 0.87 for IAP_MAL_ and 0.83 versus 0.95 for IAP_SP_, respectively).

**Table 2:** Intra-class correlation (ICC) between 3 measurements, before and after CS and immediately after and day 1 after LAVH.

	Pre-CS		Post-CS		Post-LAVH		Day 1 after LAVH	
	*IAP_MAL_*	*IAP_SP_*	*IAP_MAL_*	*IAP_SP_*	*IAP_MAL_*	*IAP_SP_*	*IAP_MAL_*	*IAP_SP_*
**N**	24	13	24	13	27	13	27	13
**ICC**	0.71	0.83	0.87	0.95	0.98	0.98	0.96	0.97
**CI**	0,52–0,86	0,64–0,94	0,76–0,94	0,86–0,98	0,92–1	0,88–1	0,93–0,98	0,93–0,99

Data are presented as ICC with an confidence interval (CI). ICC's are determined by the two-way mixed model on absolute agreement for single measures.

IAP_MAL_: IAP using the midaxillary line; IAP_SP_: IAP using the symphysis pubis; CS: caesarean section; LAVH: Laparoscopic assisted vaginal hysterectomy.

All IAP-measurements were found to be normally distributed using the Shapiro-Wilk test for normality. Both the mean IAP_MAL_ and IAP_SP_ were significantly higher before than after CS (14.0±2.6 mmHg versus 9.8±3.0 mmHg for IAP_MAL_, p<0.0001, and 8.2±2.5 mmHg versus 3.5±1.9 mmHg for IAP_SP_, p = 0.010). Based on the IAP_MAL_ values, the overall incidence of IAH (defined as IAP>12 mmHg) before CS was 83%. Within the group of IAH, incidences were 58% for grade I, 37% for grade II and 5% for grade III. None of the patients had ACS. After the CS the reported incidences were 17% and 4% respectively for grade I and II. None of the patients had IAH grade III or ACS (Table 3).

**Table 3:** Incidence of intra-abdominal hypertension (IAH) before and after caesarean section.

	IAP_MAL_				
	*Pre-CS*	*Post-CS*	*P (compared to pre-CS)*	*After LAVH*	*P (compared to pre-CS)*
**No IAH**	4 (17.4)	18 (78.3)	<0.001	21 (77.8)	<0.001
**IAH**	19 (82.6)	5 (21.7)	<0.001	6 (22.2)	<0.001
** IAH Grade I**	11 (57.9)	4 (80.0)	0.029	4 (66.7)	0.012
** IAH Grade II**	7 (36.8)	1 (20.0)	0.023	1 (16.7)	0.013
** IAH Grade III**	1 (5.3)	0	0.500	1 (16.7)	0.713

IAP is measured using the midaxillary line. Results are presented as n (%). Differences in incidence as compared with the pre-CS data are presented as p-values and were calculated with a Fischer's exact test.

IAP_MAL_: IAP using the midaxillary line; CS: caesarean section; LAVH: Laparoscopic assisted vaginal hysterectomy.

**Figure 3:**
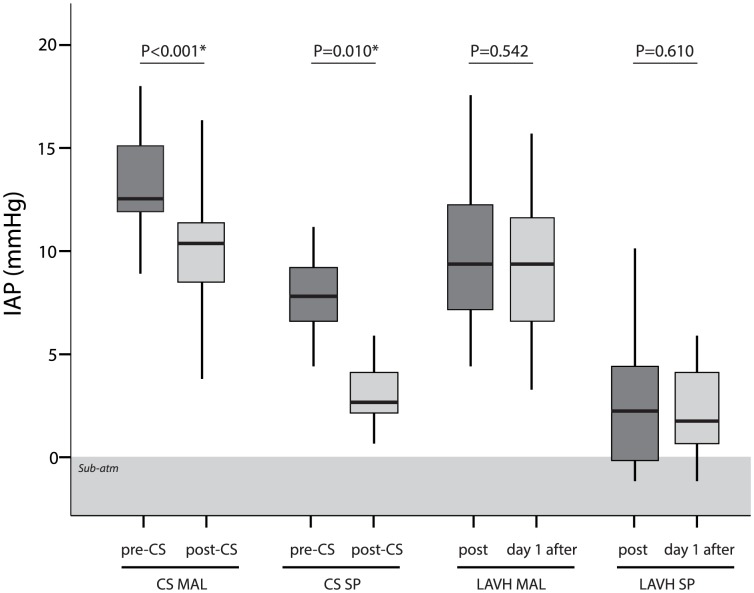
IAP before and after CS, and immediately after and day 1 after the LAVH. IAP_MAL_: IAP using the midaxillary line; IAP_SP_: IAP using the symphysis pubis; CS: caesarean section; LAVH: laparoscopic assisted vaginal hysterectomy.

The incidence of IAH regardless of grade was less in the control LAVH group (p<0.001). IAP in pregnant subjects was significantly higher than in the non-pregnant patients in the control LAVH-group day 1 after surgery (p<0.001 for both IAP_MAL_ and IAP_SP_). Moreover, there was no significant difference between both non-pregnant groups: i.e. IAP after CS and IAP day 1 after LAVH (p = 0.256 and p = 0.573 for IAP_MAL_ and IAP_SP_, respectively). In the control LAVH-group, there was no significant difference between the IAP immediately after the surgery and the IAP day 1 after the surgery (p = 0.542 for IAP_MAL_ and p = 0.610 for IAP_SP_). Results are presented in figure 3.

In all cases, IAP_SP_ was significantly lower than the associated IAP_MAL_ (p<0.001). No sub-atmospheric values were recorded using the midaxillary line. In 7 LAVH patients, a sub-atmospheric IBP was measured using the symphysis pubis as zero reference.

The pre-pregnancy weight correlated with IAP_MAL_ after CS (r = 0.41, p = 0.048), but not with IAP_MAL_ before CS. The same was observed for the patient's weight and BMI at term, just before CS: term weight correlated with post-CS IAP_MAL_ (r = 0.49, p = 0.019 for weight and r = 0.42, p = 0.049 for BMI), but not to IAP_MAL_ before CS. Next to this, IAP_MAL_ before CS correlated with fetal birth weight (r = 0.44, p = 0.035) but this did not hold true for IAP_MAL_ after the CS. A negative correlation between breech presentation and IAP_SP_ was observed (table 4). In the LAVH-group, both IAP_MAL_ and IAP_SP_ correlated significantly with the patient's weight and BMI. Correlations between the IAP and patient characteristics are presented in table 4.

**Table 4:** Correlations between the IAP and patient characteristics.

**CS-group**									
**Parameter**	*N*	IAP_MAL_ Pre-CS		IAP_MAL_ Post-CS		IAP_SP_ Pre-CS		IAP_SP_ Post-CS	
		*PCC*	*P*	*PCC*	*P*	*PCC*	*P*	*PCC*	*P*
Weight pre-pregnancy	23	0,239	0.272	0,409*	0.048*	0,335	0.241	0,383	0.176
BMI pre-pregnancy	23	0.274	0.206	0.353	0.099	0.453	0.104	0.471	0.089
Weight at term	23	0,257	0.236	0,486*	0.019*	0,384	0.175	0,410	0.145
BMI at term	23	0,299	0.165	0,415*	0.049*	0,526	0.053	0,516	0.059
Fetal birth weight	23	0,441*	0.035*	0,359	0.093	−0,043	0.880	0,062	0.832
Breech presentation	7	−0.197‡	0.368	0.042‡	0.848	−0.537‡*	0.048*	−0.401‡	0.155
Maternal age	23	−0.147	0.497	0.096	0.663	−0.100	0.746	−0.079	0.787
**LAVH-group**									
		IAP_MAL_ Post-LAVH		IAP_MAL_ 24 h		IAP_SP_ Post-LAVH		IAP_SP_ 24 h	
		*PCC*	*P*	*PCC*	*P*	*PCC*	*P*	*PCC*	*P*
Weight	27	0,700*	0.004*	0,608*	0.001*	0,526*	0.007*	0,581*	0.037*
BMI	27	0,743*	0.001*	0,676*	0.000*	0,611*	0.001*	0,648*	0.017*
Age	27	0.370	0.175	0.262	0.187	0.348	0.081	0.460	0.113

Correlations are calculated with the Pearson's product moment correlation (PCC) for numeric data, and with the point-biserial correlations coefficient (‡) for dichotomous parameters. Significant correlations are marked with an asterisk (*).

IAP_MAL_: IAP using the midaxillary line; IAP_SP_: IAP using the symphysis pubis; CS: caesarean section; LAVH: laparoscopic assisted vaginal hysterectomy.

## Discussion

### Main findings

We observed a high intra-observer correlation of IAP measurements according to the intra-bladder pressure method, with most ICC's ≥0.83. IAP was higher before than after CS in all pregnant subjects: 14.0±2.6 mmHg versus 9.8±3.0 mmHg (p<0.0001) for IAP_MAL_ and 8.2±2.5 mmHg versus 3.5±1.9 mmHg (p = 0.010) for IAP_SP_, respectively. IAP before CS is in the range of IAH in 83% of the cases for IAP_MAL_. We also found a significant correlation between the patient's weight and BMI before CS and after LAVH with the IAP_MAL_ after surgery; this was not true for the IAP_MAL_ before the CS.

### Strengths and limitations

To the best of our knowledge, only four studies have been recently published regarding IAP measurements in pregnant women and postpartum. In these papers, the intra-observer reliability of IAP measurements was not described [12], [18], [19] [20]. Our study is the first to demonstrate that one intravesical measurement according to a standard protocol is sufficient to reliably define IAP during pregnancy. This protocol eliminates the possible interference from confounders such as anaesthesia, which significantly decreases the IAP [21], and inappropriate maternal position.

The present study has some limitations. First, this study does not allow drawing conclusions on the direct effect of the pregnant uterus on the intra-bladder pressure and measured IAP-values. The observed correlation between the fetal intra-uterine positioning and the maternal IAP suggests that the gravid uterus and/or the fetal position might have a direct pressure effect on the bladder, which subsequently might influence the measured IAP. Secondly, we did not evaluate inter-observer variability since all the measurements were performed by the same investigator, and the patient's weight was not recorded after CS, excluding the calculation of the correlation between IAP after CS and the current weight. Next, the LAVH-group as a control group has an important limitation since the abdomen of the patient is inflated with CO_2_ during the LAVH. A cohort of healthy fertile women consenting for indwelling bladder catheterization would be preferable as a control group in future studies. We did not measure the IAP before the LAVH procedure but only 1 hour postoperatively, and the small sample size is also a limitation of this study. Finally, we only collected basic anthropomorphic patient data, however Sugerman states that other parameters like waist-to-hip ratio or sagittal-abdominal-diameter may be related to IAP values [22].

### Interpretation

#### Intra-observer variability

Inter- and intra-observer reliability of IAP measurements in critically ill patients has been described in several studies, resulting in varying results from fair to excellent agreement [23]–[25]. In our study, most ICC's were ≥0.83, which indicates that one single measurement according to a standard protocol seems reliable. Overall, ICC in the CS-group was lower when compared with the LAVH-group, which might partly be explained by difficulties in defining the specific zero-reference point in a pregnant woman. When compared to measurements before CS, repeatability after CS seems slightly higher, which might be due to unavoidable prenatal conditions such as Braxton Hicks contractions or fetal movements.

#### IAP before and after CS

We found a significantly higher IAP in pregnant subjects than in non-pregnant subjects (after CS and the LAVH-group). This is comparable with recently published results: Chun et al. measured a median IAP of 10.0 mmHg [12], and Fuchs mentioned a median IAP of 14.2 mmHg [20], which is in the same range as in our study.

Literature about postpartum IAP is scarce, as only three publications mention IAP measurements immediately after CS; Abdel-Razeq et al. and Fuchs et al. reported a IAP of 5.8 mmHg and around 11.1 mmHg, respectively [19], [20]. We observed a mean IAP_MAL_ of 9.8±3.0 mmHg after the CS, which is amongst their reported values and in the same range as the mean IAP_MAL_ in the LAVH-group.

Al-Khan and colleagues described a postpartum IAP of 16.4 mmHg [18]. This is higher than in our study, probably due to methodological overestimation of IAP, as is noticed by Chun et al. [10]. Nevertheless, the main finding that IAP decreases after delivery is in line with our results. This effect of an abdominal tumour or an enlarging uterus has been simulated by Bloomfield in an experimental study in dogs [26]. They demonstrated that a growing intra-abdominal volume causes a gradual increase in IAP by progressively inflating an intra-abdominal balloon. The increasing volume was associated with a significant increase in IAP that resolved with balloon deflation [26].

#### Correlation between IAP and body anthropomorphism

The study of Abdel-Razeq described a significant correlation with IAP after CS and the pre-pregnancy BMI [19], which is in line with the results of our study as we found a significant correlation between the pre-pregnancy weight and IAP_MAL_ after CS. Moreover, the fact that the weight and BMI before the CS correlates well with IAP_MAL_ after CS, but not with the IAP_MAL_ before CS, suggests that IAP is indeed determined by the patient's weight, but only when the fetus-factor is eliminated. The intra-uterine fetus seems to influence the maternal IAP, the IAP measurement using the FMLV method, or both. The hypothesis that the fetus influences the maternal IAP is supported by the significant correlation in our study between the fetal birth weight and the IAP_MAL_ before CS, but not with the IAP_MAL_ after CS, and this can be explained by the increase in intra-abdominal volume by the fetus and the relation between IAP as determined by the abdominal wall compliance.

#### IAP and maternal complications

Maternal venous Doppler studies illustrated a shift from hepatic vein triphasic patters in early pregnancy to flat patterns at term, which is similar to the hepatic vein changes following the Valsalva or an intra-abdominal tumour [27], [28]. Also, an increase in the femoral venous pressure during pregnancy has been described [29]. Next to this, a different pattern in Doppler waves in uncomplicated pregnancies versus hypertensive gestational diseases is described [30], [31]. Sugerman hypothesized that an excessively high IAP during pregnancy compresses the venous system, which might contribute to symptoms known as preeclampsia and HELLP-syndrome [11]. Consequently, there might be a relation between the augmented IAP and the development of venous insufficiency during pregnancy, and the onset of planar oedema in the third trimester [32]. Bloomfield observed in experimental conditions that an increasing IAP goes along with an increasing systolic and diastolic blood pressure [26]. In this context, it could be hypothesized that a relation exists between IAP and gestational complications, such as preeclampsia and more in particular: late-onset preeclampsia which usually presents less aggressive than early onset preeclampsia, mostly in obese women with large uteri [33]. In this respect, it could also be hypothesised that the curative effect of delivery upon the symptoms of preeclampsia might not always be a primary result of removal of the placenta, and that a decline of IAP following delivery might also have a role to play. Based on this, studies on the correlation between the venous Doppler pattern, IAP and gestational complications are a goal for further research.

## Conclusion

Measurements of IAP in pregnant women using the FMLV are highly reliable and reproducible. After CS, IAP significantly declines from hypertensive to normal non-pregnant values. It can be stated that pregnancy seems a physiological status of intra-abdominal hypertension. Birth weight and fetal presentation influence IAP in term pregnant women, which might partly explain the wide inter-individual differences of prenatal IAP.
